# Cellular Functions and Gene and Protein Expression Profiles in Endothelial Cells Derived from Moyamoya Disease-Specific iPS Cells

**DOI:** 10.1371/journal.pone.0163561

**Published:** 2016-09-23

**Authors:** Shuji Hamauchi, Hideo Shichinohe, Haruto Uchino, Shigeru Yamaguchi, Naoki Nakayama, Ken Kazumata, Toshiya Osanai, Takeo Abumiya, Kiyohiro Houkin, Takumi Era

**Affiliations:** 1 Department of Neurosurgery, Hokkaido University Graduate School of Medicine, Sapporo, Japan; 2 Department of Cell Modulation, Institute of Molecular Embryology and Genetics, Kumamoto University, Kumamoto, Japan; University of Kansas Medical Center, UNITED STATES

## Abstract

**Background and purpose:**

Moyamoya disease (MMD) is a slow, progressive steno-occlusive disease, arising in the terminal portions of the cerebral internal carotid artery. However, the functions and characteristics of the endothelial cells (ECs) in MMD are unknown. We analyzed these features using induced pluripotent stem cell (iPSC)-derived ECs.

**Methods:**

iPSC lines were established from the peripheral blood of three patients with MMD carrying the variant *RNF213 R4810K*, and three healthy persons used as controls. After the endothelial differentiation of iPSCs, CD31^+^CD144^+^ cells were purified as ECs using a cell sorter. We analyzed their proliferation, angiogenesis, and responses to some angiogenic factors, namely VEGF, bFGF, TGF-β, and BMP4. The ECs were also analyzed using DNA microarray and proteomics to perform comprehensive gene and protein expression analysis.

**Results:**

Angiogenesis was significantly impaired in MMD regardless of the presence of any angiogenic factor. On the contrary, endothelial proliferation was not significant between control- and MMD-derived cells. Regarding DNA microarray, pathway analysis illustrated that extracellular matrix (ECM) receptor-related genes, including integrin β3, were significantly downregulated in MMD. Proteomic analysis revealed that cytoskeleton-related proteins were downregulated and splicing regulation-related proteins were upregulated in MMD.

**Conclusions:**

Downregulation of ECM receptor-related genes may be associated with impaired angiogenic activity in ECs derived from iPSCs from patients with MMD. Upregulation of splicing regulation-related proteins implied differences in splicing patterns between control and MMD ECs.

## Introduction

Moyamoya disease (MMD) is a slow, progressive steno-occlusive disease arising in the terminal portions of the cerebral internal carotid artery (ICA). An abnormal vascular network subsequently develops at the base of the brain as collateral circulation against decreased blood supply.[1] Histopathological studies revealed remarkable intimal thickening, waving of the internal elastic lamina, and medial thinning at the terminal portion of ICA in patients with MMD.[2] Several pathogenic mechanisms, including inflammation,[3] upregulation of various angiogenic factors,[2, 4, 5] and abnormalities of endothelial progenitor cells (EPCs),[6] have been hypothesized.

Kim et al. revealed that the angiogenic activity of EPCs in patients with MMD was impaired and this indicated that the phenotype of EPCs could be a disease-specific in vitro model.[6] However, they had difficulty in conducting reproductive and comprehensive studies using EPCs because of the limited proliferative activity of the primary cells. In 2007, Yamanaka et al. firstly reported the establishment of human-induced pluripotent stem cells (iPSCs),[7] and at present, iPSCs are recognized to be useful for studying the mechanism of diseases within specific genetic backgrounds as well as advancing regenerative medicine.

Recently, ring finger protein 213 (*RNF213*) was identified as a susceptibility gene for MMD in East Asians via a genome-wide linkage and association study. Mutation in *RNF213* was found in 95% of patients with familial MMD, 73% of patients with non-familial MMD, and 1.4% of controls.[8] After these discoveries, research focusing on the biological effect of mutant *RNF213* has progressed.

Hitomi et al. first established MMD-specific iPSCs carrying *RNF213 R4810K* from skin fibroblasts and differentiated these cell lines into endothelial cells (ECs).[9] They reported that these cells featured impaired angiogenesis and concluded that the iPSCs-derived-ECs (iPSECs) can be regarded as an in vitro model of MMD because these ECs have a similar phenotype as those EPCs obtained from patients with MMD.

In the present study, we established iPSC lines from the peripheral blood mononuclear cells (MNCs) of patients with MMD carrying *RNF213 R4810K*, and differentiated these cell lines into ECs. To elucidate novel aspects of the pathogenesis of MMD, we investigated cellular functions such as proliferation and angiogenesis via comprehensive gene and protein expression analysis in ECs generated from iPSCs.

## Materials and Methods

### Subjects

MMD-specific iPSCs were derived from three unrelated patients with familial MMD. The gender and age of subjects with MMD was as follows: MMD 1, 39-year-old male; MMD 2, 48-year-old female; and MMD 3, 36-year-old female. Three normal subjects were recruited as controls (Control 1, 48-year-old male; Control 2, 54-year-old male; Control 3, 48-year-old male). Their diagnoses were based on criteria from the Japanese Research Committee on MMD (Ministry of Health, Labour and Welfare, Japan). Patients and normal subjects were recruited with written informed consent which was approved by the institutional review board of Hokkaido University Hospital and Kumamoto University.

All experimental procedures of human samples were approved by the ethics committees, “Ethics Committee of Hokkaido University Hospital”, “Ethics committee for Epidemiological and General Research at the Faculty of Life Science, Kumamoto University”, “Ethics committee for Human genome and Gene analysis Research at the Faculty of Life Sciences, Kumamoto University” and “Ethics committee for clinical research and advanced medical technology, Kumamoto University” (approval numbers 012–0317, 318, 153 and 1018, respectively). All animal experimentation was conducted at Kumamoto University after receiving approval from the Committee for Ethics on Animal Experiments of Kumamoto University (approval numbers A25-084).

### iPSCs generation

MNCs were isolated by gradient centrifugation with Ficoll-Qaque, and then they were activated and expanded in KBM502 medium (Kohjin Bio Co., Saitama, Japan) on anti-CD3 antibody-coated dishes (eBioScience, San Diego, CA). iPSCs were generated from activated MNCs as described previously.[10] In brief, 5 × 10^5^ MNCs were infected with Sendai virus carrying *OCT3/4*, *SOX2*, *KLF4*, and *c-MYC* at a multiplicity of infection of 10. Sendai virus was prepared as described previously.[11] After 2 days of culture, the infected cells were seeded at 2 × 10^4^ cells per 10 cm dish on mitomycin C (MMC)-treated mouse embryonic fibroblasts (MEFs). On the next day, the medium was replaced with iPS cell medium. From 15 to 17 days after infection, the colonies were selected and expanded on MEFs with iPS medium.

### Endothelial differentiation of iPSCs

Endothelial differentiation was performed as described previously with some modifications.[12] The iPSCs at subconfluency were detached using CTK solution consisting of 0.1 mg/ml collagenase IV (Invitrogen), 0.25% trypsin (Invitrogen), 0.1 mM CaCl2 (Nacalai tesque) and 20% KSR and seeded onto Matrigel-coated dishes at a ratio of 1:5 to 1:10. We treated iPSCs with 50 ng/mL bone morphogenetic protein 4 (BMP4; R & D Systems, Minneapolis, MN) and 50 ng/mL basic fibroblast growth factor (bFGF; Wako, Osaka, Japan) for the first 24 h; 40 ng/mL vascular endothelial growth factor (VEGF; Invitrogen, Waltham, MA) and 50 ng/mL bFGF for 2 days; and 40 ng/mL VEGF, 50 ng/mL bFGF, and 20 μmol/L SB431542 (Miltenyi Biotec, Teterow, Germany) for 3–4 days. On days 6–7, ECs were purified using FITC-conjugated anti-CD31 antibody (1 μg/1 × 10^6^ cells, WM59; BioLegend, San Diego, CA) and APC-conjugated anti-CD144 antibody (0.5 μg/1.0 × 10^6^cells; 16B1, eBioscience) with a FACSAria III (BD Biosciences, San Jose, CA).

### Cell culture

iPSCs were maintained on MMC-treated MEFs in iPS medium containing DMEM/F12 (Wako) supplemented with 20% KnockOut Serum Replacement (Invitrogen), 2 mmol/L l-Alanyl-l-Glutamine (Wako), 0.1 mmol/L monothioglycerol (Wako), 0.5% penicillin and streptomycin (Nacalai Tesque, Kyoto, Japan) and 5 ng/ml basic fibroblast growth factor (Wako). The iPSECs were maintained on collagen I-coated dishes with HuMedia-EB2 medium (KURABO, Japan), supplemented with 20 ng/ml VEGF (R & D Systems), 25 ng/ml bFGF, 0.5% penicillin and streptomycin, and 10% fetal bovine serum (FBS).

### CCK8 cell proliferation assay

Cell proliferation was analyzed using CCK8 (Dojindo, Kumamoto, Japan) according to the manufacturer’s instructions. iPSECs at subconfluence were serum- and growth factor-starved overnight before the experiment. Cells were seeded at 5 × 10^3^ cells per well in 96-well plates. After attachment (at 0 and 72 h), the cells were treated with 10 μL of WST-8 dye and incubated at 37°C for 2 h. To estimate the proliferative cell numbers, absorbance was determined at a wavelength of 450 nm using a microplate reader (TECAN). To analyze population doubling time (DT), the iPSECs were cultured with HuMedia-EB2 medium supplemented with 20 ng/ml VEGF, 25 ng/ml bFGF, and 10% FBS and the cells were counted after cell attachment and at 24 h and 48 h using CCK8. Population doubling time was calculated using the formula: DT = duration × log(2)/(log N−logN0), where N and N0 are the numbers of cells at counting and initial plating.

### Tube formation assay

The iPSECs at subconfluence were serum- and growth factor-starved overnight. Then the iPSECs were detached, suspended in HuMedia without serum or angiogenic factors, and seeded onto Matrigel-coated 96-well plates at a density of 5000 cells/well. These cells were then incubated with or without the indicated growth factors (VEGF, bFGF, BMP4, and TGF-β at 50 ng/ml). After 12 h of culture, digital images of tube formation were captured by an inverted microscope (Olympus, Tokyo, Japan) using a 4 × objective lens. For quantification, total tube length was automatically measured by the ImageJ tool program (National Institutes of Health, Bethesda, MD).

### DNA microarray analysis

Total RNA was extracted from iPSECs with an RNeasy Mini Kit (Qiagen, Venlo Netherlands). Total RNA samples were reverse-transcribed, amplified, and labeled using a GeneChip^®^3′ IVT Express Kit (Affymetrix, Santa Clara, CA). The cRNA samples were hybridized to an Affymetrix Human Genome U133 Plus 2.0 array. For scanning, an Affymetrix GeneChip Scanner 3000 7G was used. Expression values were determined using GeneChip Command Console Software and Expression Console Software. Raw data was imported to GeneSpring GX software (Agilent Technologies). Unsupervised clustering was conducted using the following steps: Probes that did not have a gene symbol were excluded; probes with a standard deviation from the expression level of more than 0.5 were selected, and probes with a minute expression level and a signal intensity of less than zero in more than four samples were excluded.

### RT-PCR

Total RNA was purified with Sepasol Super G reagent (Nacalai Tesque). Total RNA was transcribed to DNA with Superscript III (Invitrogen) and random primers (Invitrogen). RT-PCR was conducted using QuickTaq (Toyobo, Osaka, Japan), according to the manufacturer’s instructions. The sequences of primers and amplification conditions for the detection of pluripotent markers were designed as described previously. The primers used for *OCT3/4*, *SOX2*, *KLF4*, and *c-MYC* were designed to detect the expression of endogenous genes but not of transgenes. To detect the SeV genome, nested RT-PCR was performed. To analyze the result of microarray, the following primer sets were used: Integrin β3 (*ITGB3*); forward, 5′-TGTACCACGCGTACTGACAC-3′, reverse, 5′-CACTTCTCACAGGTGTCCCC-3′; integrin β8 (*ITGB8*); forward 5′-CCCGTGACTTTCGTCTTGGA-3′, reverse 5′-GGGGAGGCATGCAGTCTAAA-3′.

### DNA isolation and Sanger sequencing

MNCs were treated with Lysis buffer (10 mmol/L Tris-HCL pH7.5; 10 mmol/L EDTA; 10 mmol/L NaCl; 1 mg/mL proteinase K, 0.5% SDS). Genomic DNA was precipitated with ice-cold 75 mM NaCl in ethanol and suspended with 50 μL of TE buffer (10 mmol/L Tris-HCl; 1 mmol/L EDTA, pH 8.0).

Mutation of *RNF213* (*p*.*R4810K*: *rs112735431*, *G > A*) was analyzed by direct sequencing. The sequence reactions were performed using a Big Dye Terminator cycle sequencing kit (Life Technologies, Carlsbad, CA) and analyzed using an ABI PRISMTM 310 Genetic Analyzer (Applied Biosystems, Waltham, MA). The sequences of primers for detecting *R4810K* in *RNF213* were as follows: forward, 5′-CAAGCTCCACGCTGCATCAC-3′, reverse, 5′-CACCCTGTTCCCCTATGCAG-3′.

### Western blotting

Whole cell extracts were isolated using a Minute Protein Extraction Kit (Invent Biotechnologies, Eden Prairie, MN) according to the manufacturer’s instructions. The protein extract (10 μg) was separated by electrophoresis using 4–12% sodium dodecyl sulfate polyacrylamide gels and transferred to nitrocellulose membranes using iBlot Systems (Invitrogen). After electroblotting, the membrane was blocked in 2% bovine serum albumin at room temperature for 30 min and probed with primary antibody at 4°C overnight in Can Get Signal Solution-1 (Toyobo). The dilution ratios of the primary antibodies were as follows: Rabbit anti-integrin β3 (1:1000; Cell Signaling Technology, Danvers, MA) and anti-alpha-tubulin (1:2000; Cell Signaling Technology). The membrane was incubated for 1 h at room temperature in Can Get Signal Solution-2 with horseradish peroxidase-conjugated secondary antibody (1:2000, Cell Signaling Technology). Immunoreactive bands were detected using an enhanced chemiluminescence detection system according to the manufacturer’s protocol.

### Proteomics

Total proteins were extracted from iPSECs with lysis buffer (5 mol/L urea, 2 mol/L thiourea, 2% CHAPS, 2% SB3-10, 1% DTT). Extracted proteins were separated using Immobiline DryStrip (pH 4–7, 24 cm; GE Healthcare Bio-Sciences, Pittsburgh, PA) for the first dimension, and SDS-PAGE in 9–18% acrylamide gradient gel for the second dimension, followed by staining with SYPRO Ruby protein gel stain (S21900; Invitrogen). The stained gels were scanned with Molecular Imager FX (Bio-Rad Laboratories, Hercules, CA) and were analyzed using the Image Master Platinum (GE Healthcare Bio-Sciences). To normalize the spot volume values in each gel, the raw quantity of each spot was divided by the total volume of all spots included in the same gel (% volume). The spots significantly differentially expressed with a difference of more than 1.5-fold (*P* < 0.05) between MMD, and control samples were determined and excised from the gels. The tryptic digests of excised proteins were assessed by MS/MS ion search to identify proteins from the peptide sequence database. Protein identification was accepted when the matching scores were significant at *P* < 0.05 as based on the MOWSE score using the Mascot search engine.

### Flow cytometry and cell sorting

For CD144 and CD31 staining, the differentiating iPS cells were dissociated with TrypLE on day 6. The dissociated cells were collected into a 15 ml conical tube with 10% FBS/PBS and the cell number was counted. The cells were centrifuged and supernatant was discarded. FITC-conjugated anti-human CD31 antibody (BD Biosciences) and APC-conjugated anti-human CD144 antibody (eBioscience) were added to the cell suspensions at the concentration of 1 μL/1×10^6^ cells. After incubated with the antibodies for 20 min, the cells were washed twice with 10% FBS/PBS and resuspended. The concentration of the cell pellet was adjusted to 5×10^6^ /ml with 10% FBS/PBS. The cells were analyzed and sorted by FACS Aria III (BD Biosciences) at flow rates ≦ 2000 events/second. The APC antibody was excited at 633 nm and collected at 660/20 nm. The FITC antibody was excited at 488 nm and collected at 530/30 nm.

### Immunocytochemistry

Cells were rinsed in PBS and fixed in 4% paraformaldehyde for 10 min. After blocking with PBS containing 1% BSA, primary antibodies were added in blocking solution overnight at 4°C. The cells were then washed in PBS three times and incubated with the secondary antibodies in blocking solution for 1 h at room temperature. The primary antibodies used in this experiment were as follows: Anti-CD144 (1:100, clone 55-7H1; BD Pharmingen); anti-von Willebrand factor (vWF, 1:100, Clone F8/86; Dako, Glostrup, Denmark); and anti-VEGFR2 (1:200, 55B11, Cell Signaling Technology).

### Teratoma formation

All experimental procedures conformed to the animal use guidelines of the Committee for Ethics on Animal Experiments of Kumamoto University (approval numbers A25-084). MMD-iPSCs were collected by CTK solution and injected intratesticularly into NOD/Scid/Jak3 KO mice. The mice were purchased from Kyudo Co., Ltd (Kumamoto, Japan). Animals received minimal handling and procedures were performed under diethyl ether anesthesia. Mice were housed in wire topped clear perspex cage and given ad libitum access to food and water. The light cycle was a standard 12 light: 12 dark, commencing with light at 07:00. Animals were monitored and assessed for health and condition daily. After teratomas become palpable, mice were euthanized by cervical dislocation. Tumor samples were collected, fixed in 4% paraformaldehyde, and processed for histological analysis. No animals became severely ill or died prior to the experimental endpoint.

### Statistical analysis

Every result is presented as the mean ± standard deviation. Student’s *t*-test was used to analyze the difference between the means in each group. Statistical significance was accepted at the 95% confidential level (*P <* 0.05). All experiments were repeated at least three times, and representative data are shown.

## Results

### Characterization of established iPSCs

**Fig 1:**
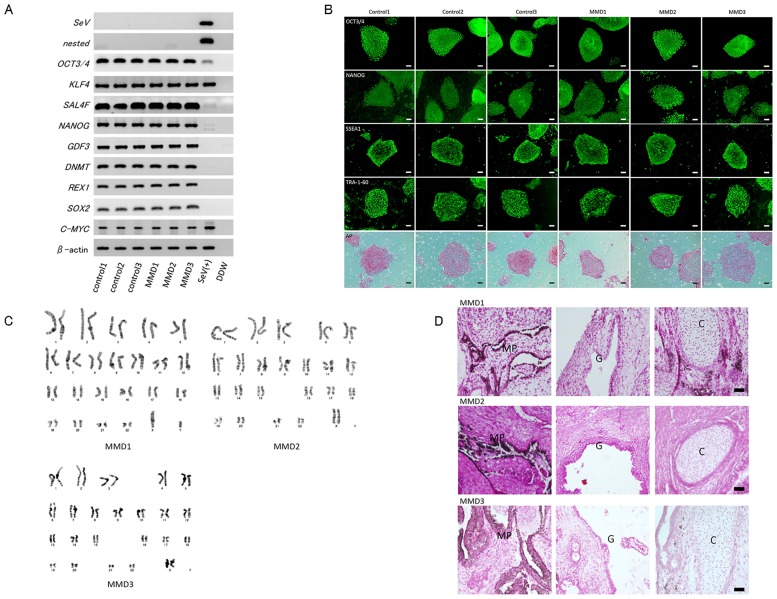
Characterization of human-induced pluripotent stem cells (iPSCs) from patients with moyamoya disease (MMD) and control subjects. Panel A shows the results of RT-PCR analysis of the Sendai virus and human embryonic stem (ES) cell markers in each iPSC line. Panel B shows the immunoreaction of ES cell markers *OCT3/4*, *NANOG*, *SSEA1*, and *TRA-1-60* and the reaction for alkaline phosphatase staining in all iPSC colonies. Scale bars: 100 μm. Panel C shows the representative photomicrographs of karyotype analysis in each MMD-iPSC line. Panel D shows the representative photomicrographs of the formation of teratoma in each MMD-iPSC line. “G”: Glandular structure (endoderm), “C”: Cartilage (mesoderm), and “MP”: Melanin pigment (ectoderm). Scale bars: 100 μm.

All three patients had the common *R4810K* mutation of *RNF213*, and control subjects had no *R4810K* mutation in *RNF213*. In all cell lines, expression of pluripotent markers and positive findings for ALP staining were noted (Fig 1A and 1B). Nested RT-PCR analysis illustrated that MMD-iPSC lines were negative for the SeV genome (Fig 1A). Karyotype analysis revealed that the MMD-iPSC lines had a normal karyotype (Fig 1C). We confirmed the differentiation potential of MMD-iPSCs into three germ layers by teratoma formation (Fig 1D).

### Endothelial differentiation of iPSCs

**Fig 2:**
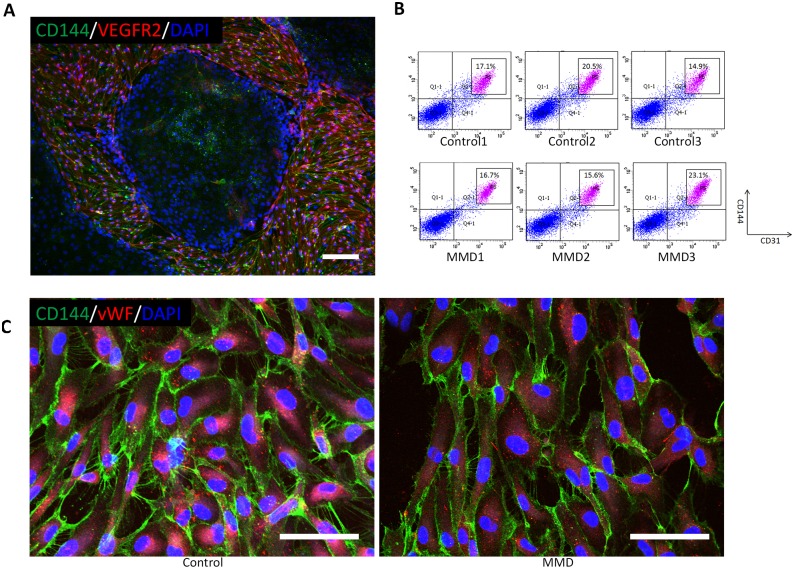
Endothelial differentiation of induced pluripotent stem cells (iPSCs). Panel A shows representative photomicrographs of immunocytochemistry (CD144: Green, VEGFR2: Red, DAPI: Blue) in iPSCs 6 days after endothelial differentiation. Scale bar: 500 μm. Panel B shows the results of FACS analysis of CD144 and CD31 in the differentiated iPSCs. Sorted cells are gated and indicated as P5. Panel C shows the representative photomicrographs of immunocytochemistry for CD144 (green) and von Willebrand factor (vWF) after the purification and expansion of endothelial cells. Left: Control, right: MMD. Scale bar, 100 μm.

In every iPSC line, immunocytochemistry identified some CD144^+^VEGFR2^+^ cells 6 days after endothelial differentiation (Fig 2A). The FACS analysis also illustrated that their population consisted of 16–23% CD31^+^CD144^+^ cells (Fig 2B). After the CD31^+^CD144^+^ cells were purified and expanded, they were regarded as iPSCs because of their positive expression of endothelial markers such as CD144 and vWF.

### Proliferation, angiogenesis, and response of iPSECs to angiogenic factors

**Fig 3:**
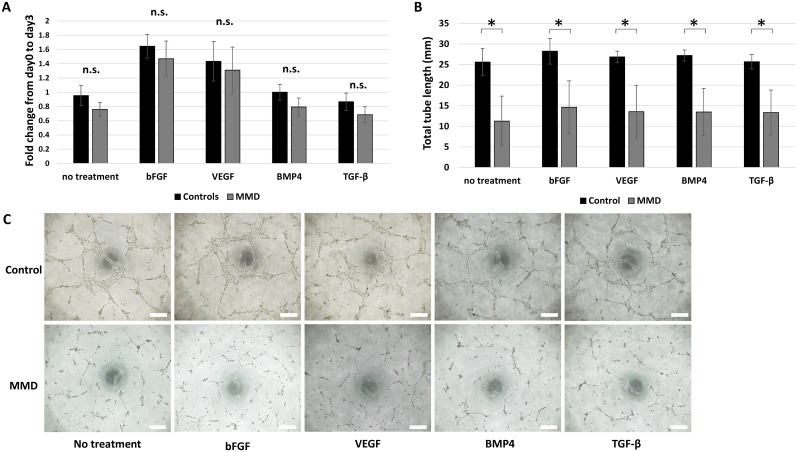
Cellular functions of iPSCs-derived endothelial cells (iPSECs). Panels A shows the results of CC8 cell proliferation assay (A) after 3 days of culture. Panel B shows that representative photomicrographs of the tube formation assay. Panel C shows the quantitative analysis of total tube length. *: *P* < 0.05, error bars: SD, n.s.: No significance. Scale bar: 500 μm.

The mean population doubling time of iPSECs calculated at 48 h was 19.0 ± 0.63 h in the control population and 21.8 ± 3.75 h in the MMD population; there was no significant difference between these. Exposure to angiogenic factors for 72 h did not affect proliferation (Fig 3A). On the contrary, the *in vitro* angiogenesis assay demonstrated that iPSECs derived from patients with MMD carrying the R4810K mutation exhibited significantly reduced total tube length in the presence or the absence of angiogenic factors (*P* < 0.05, Fig 3B and 3C).

### Downregulation of ECM receptor-related genes in iPSCs

**Fig 4:**
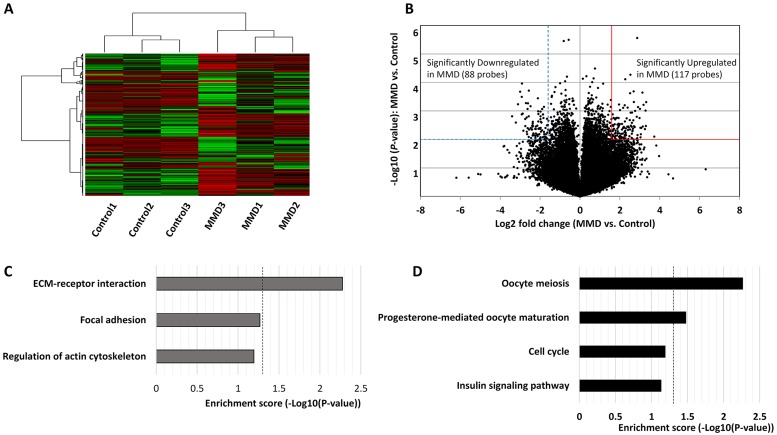
The result of DNA microarray analysis. Panel A shows unsupervised clustering analysis of moyamoya disease (MMD)—and control—iPSECs. Panel B shows the volcano plot of all probes. The broken blue line and continuous red line denote significantly downregulated and upregulated genes in MMD-iPSECs, respectively (fold change >3, *P* < 0.01). Panels C and D show the significantly downregulated and upregulated genes in pathway analyses of MMD-iPSECs, respectively. Dotted line indicates the significance level (*P* < 0.05).

The microarray expression data (after RMA background correction, 75 percentile normalization, and log2 transformation) are provided in supplementary materials (S1 Dataset). DNA microarray analysis demonstrated that the steps of unsupervised hierarchical clustering extracted 1527 of 54,680 probes and distinguished MMD cells from control cells clearly (Fig 4A). Using volcano plots, we found that 88 probes were downregulated by less than 1/3 fold (S2 Dataset) and 117 genes were upregulated by more than 3 fold (S3 Dataset) in MMD-iPSCs (*P <* 0.01, Fig 4B). KEGG pathway analysis of these downregulated genes illustrated that the enrichment score of ECM receptor-related genes, such as glycoprotein IX, heparan sulfate proteoglycan 2, *ITGB3*, and *ITGB8*, were significantly higher in MMD-iPSECs than in control cells (*P <* 0.01, Fig 4C). In the list of upregulated genes, oocyte meiosis-related genes, such as mitotic arrest-deficient 2, budding uninhibited by benzimidazoles 1 homolog (*BUB1*), calmodulin-like 6, and *SECURIN*, were significantly enriched in MMD iPSECs (*P* < 0.01). In addition, progesterone-mediated oocyte maturation-related genes, such as *BUB1* and mitogen-activated protein kinase 10, were also significantly enriched (*P* < 0.05, Fig 4D).

### Downregulation of *ITGB3* in MMD-iPSECs

**Fig 5:**
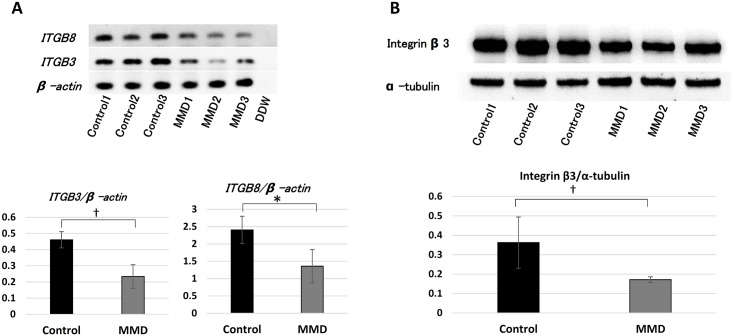
The results of RT-PCR analysis and Western blotting in iPSECs. Panel A shows the photographs of RT-PCR for integrin β3 (*ITG-β3*) and *ITG-β8* in MMD-iPSECs (*upper*) and the quantitative data (*middle*: *ITG-β3* and *lower*: *ITG-β8*, *P < 0.05, †*P* < 0.02). β-actin was used to normalize loading variations. Panel B shows the photographs of Western blotting for interin β3 in MMD-iPSECs (*upper*) and the quantitative data (*lower*, †*P* < 0.02). α-tubulin was used to normalize loading variations.

We performed RT-PCR analysis of *ITGB3* and *ITGB8* in iPSECs, because DNA microarray analysis indicated that they were downregulated significantly. RT-PCR revealed that expression of *ITGB3* and *ITGB8* in MMD-iPSECs was significantly downregulated (Fig 5A). Concerning the protein expression level, Western blotting analysis also demonstrated that integrin β3 was significantly downregulated in MMD-iPSECs (Fig 5B).

### Difference of protein expression between MMD- and control-iPSECs

**Fig 6:**
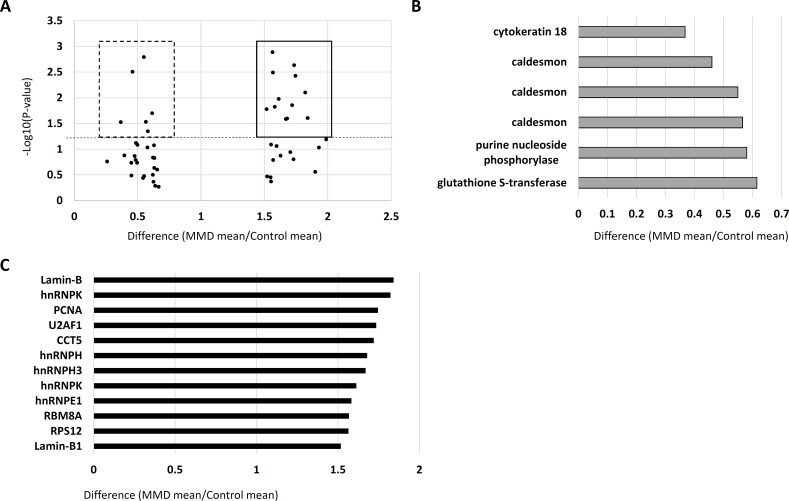
The results of proteomics. Panel A shows the protein spots with differences in expression of more than 1.5-fold or less than 0.67-fold between control- and moyamoya disease-iPSECs. Squares denote significantly upregulated proteins, and dashed squared denote significantly downregulated proteins in MMD-iPSECs. X-axis: Difference, Y-axis: P-value, and dashed line: P-value of 0.05. MS-MS ion search detected four proteins in (B) that were significantly downregulated in MMD-iPSECs and 10 proteins in (C) that were significantly upregulated in MMD-iPSECs.

In proteomics analysis, nearly 2000 protein spots were detected in each gel. Gel analysis revealed that 18 protein spots were differentially expressed by at least 1.5-fold between MMD and control cells. In detail, there were 12 protein spots with increased expression and 6 spots with decreased expression in MMD-iPSECs (Fig 6A). As shown in Fig 6B, the expression of cytokeratin 18, caldesmon, purine nucleoside phosphorylase, and glutathione S-transferase, was significantly decreased in MMD-iPSECs. Caldesmon was detected in three spots due to the existence of protein isoforms. On the contrary, the expression of lamin-B, heterogeneous nuclear ribonucleic protein K (hnRNPK), proliferating cell nuclear antigen, U2 small nuclear RNA auxiliary factor 1, chaperonin containing TCP1 subunit 5, hnRNPH1, hnRNPH3, hnRNPE1, RNA binding motif protein 8A, and ribosomal protein S12 were significantly increased in MMD-iPSECs (Fig 6C). Lamin-B and hnRNPK were detected in two spots due to the existence of protein isoforms.

## Discussion

In this study, we reported the generation of iPSC lines from peripheral blood MNCs of patients with MMD and their endothelial differentiation for the first time, as opposed to the use of fibroblasts from patients with MMD.[9] We observed that angiogenic activity was significantly reduced in the MMD-iPSECs even in the absence of angiogenic factors. However, there was no significant difference in proliferative activity. We also found using DNA microarray that four ECM receptor-related genes were significantly downregulated in MMD-iPSECs. Among these, we focused on ITGβ3, which was also downregulated at the protein level. Furthermore, proteomics analysisshowed that the expression of four cytoskeleton-related proteins was reduced and the expression of five types of hnRNP proteins was increased in MMD-iPSECs.

It is known that the tube formation of cultured ECs is induced by ECM gel and enhanced by angiogenic factors. Thus impaired tube formation suggests that the response to ECM is downregulated in MMD-iPSECs. ITG-β3, one of the representative ECM receptor-related proteins, is expressed abundantly in ECs, and it contributes to angiogenesis.[13] The downregulation of ITG-β3 may therefore be associated with impaired tube formation in MMD-iPSECs. Furthermore, the downregulation of ECM receptor-related genes in MMD-iPSECs may be associated with the downregulation of cytoskeleton-related proteins because the ECM receptor can regulate the cytoskeleton via Rho-family GTPases.[14]

In the present study, no angiogenic factors increased angiogenesis in iPSECs. Previous studies have reported an abnormal response to some angiogenic factors in smooth muscle cells in patients with MMD.[15] Interestingly, it has reported that various angiogenic factors such as bFGF, VEGF, and TGF-β were upregulated in specimens from patients with MMD, including serum, cerebrospinal fluid, and vascular wall tissue from patients with MMD.[2, 4, 5, 16] The author suggested that impaired angiogenic activity would upregulate the release of some angiogenic factors, but the negative feedback mechanism did not work sufficiently in patients with MMD because of abnormal responses to the angiogenic factors.

We found from the proteomics analysis that some splicing regulators of pre-mRNA, such as hnRNP proteins, were upregulated in MMD-iPSECs. These proteins play an important role in regulating alternative splicing[17] and alternative splicing is known to be important for increasing proteomic diversity. It has been suggested that cell-type specific splicing patterns are essential for the functions and characteristics peculiar to each cell type, and disruption of normal splicing patterns is known to cause many diseases.[18] In addition, past studies have reported that overexpression of these proteins altered the splicing pattern of targeted genes.[19] The upregulation of the splicing regulators in MMD-iPSECs may induce the altered splicing patterns that affect endothelial functions in patients with MMD. Future research into the global splicing pattern in MMD-specific ECs may clarify this new aspect of the pathogenesis of MMD.

There were some limitations to our study. We did not investigate the relation between abnormal RNF213 and differentially expressed genes or proteins. In addition, we did not investigate how these differentially expressed genes or proteins contribute to impaired tube formation in MMD-iPSECs.

Little is known about the pathogenesis of MMD even after discovery of RNF213 as the susceptibility gene for MMD. Because of the low penetrance of this mutation, environmental factors are considered to be important for promoting the pathogenesis of MMD. Houkin et al. proposed a double hit hypothesis in which, in addition to the mutation in RNF213, some triggers such as hemodynamic stress, ischemia, infection or immune disorder are essential in starting the first step in the pathological process of the disease.[20] Although we showed in this study the nature of endothelial cells of MMD under ordinary culture conditions, future analysis of the cell function of MMD under shear stress or hypoxic conditions may clarify the mechanism that leads to the development of MMD.

In conclusion, our research demonstrated the downregulation of extracellular matrix-related genes and upregulation of splicing regulating proteins in MMD-iPSECs. Further research is needed to clarify the pathological role of these differentially expressed genes and proteins in the development of MMD.

## Supporting Information

S1 Dataset Annotation for all probes sets in the Affymetrix Human Genome U133 Plus 2.0 array. (XLSX)

S2 Dataset 88 genes significantly downregulated MMD-iPSCs. (XLSX)

S3 Dataset 117 genes significantly upregulated in MMD-iPSCs. (XLSX)
